# Expansion microscopy in honeybee brains for high-resolution neuroanatomical analyses in social insects

**DOI:** 10.1007/s00441-023-03803-4

**Published:** 2023-07-08

**Authors:** Nadine Kraft, Thomas S. Muenz, Sebastian Reinhard, Christian Werner, Markus Sauer, Claudia Groh, Wolfgang Rössler

**Affiliations:** 1 Department of Behavioral Physiology and Sociobiology (Zoology II), Theodor-Boveri-Institute, Biocenter, Julius Maximilian University, Würzburg, 97074 Germany; 2 Department of Biotechnology and Biophysics, Theodor-Boveri-Institute, Biocenter, Julius Maximilian University, Würzburg, 97074 Germany

**Keywords:** Mushroom bodies, Insect, Expansion microscopy, Microglomeruli, Structural synaptic plasticity

## Abstract

The diffraction limit of light microscopy poses a problem that is frequently faced in structural analyses of social insect brains. With the introduction of expansion microscopy (ExM), a tool became available to overcome this limitation by isotropic physical expansion of preserved specimens. Our analyses focus on synaptic microcircuits (microglomeruli, MG) in the mushroom body (MB) of social insects, high-order brain centers for sensory integration, learning, and memory. MG undergo significant structural reorganizations with age, sensory experience, and during long-term memory formation. However, the changes in subcellular architecture involved in this plasticity have only partially been accessed yet. Using the western honeybee *Apis mellifera* as an experimental model, we established ExM for the first time in a social insect species and applied it to investigate plasticity in synaptic microcircuits within MG of the MB calyces. Using combinations of antibody staining and neuronal tracing, we demonstrate that this technique enables quantitative and qualitative analyses of structural neuronal plasticity at high resolution in a social insect brain.

## Introduction

**Fig. 1:**
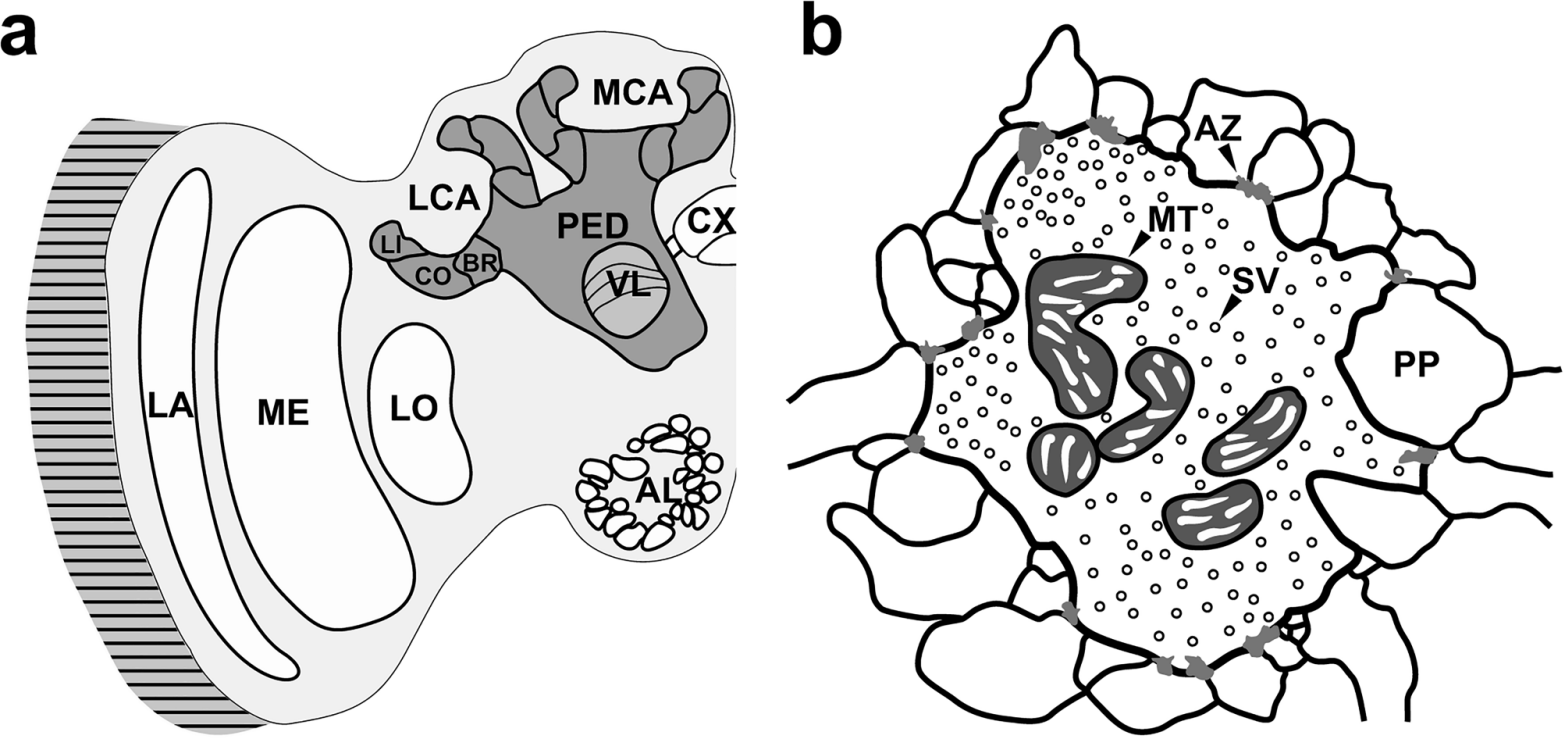
Structure and organization of mushroom bodies (MBs) and microglomeruli (MG).** a** Right hemisphere of a honeybee brain. The MB (dark gray) is formed by two large, cup-shaped major sensory input regions –the medial (MCA) and lateral calyx (LCA)– and the stalk-like peduncle (PED), which connects to the vertical lobe (VL), one of the MB’s major output regions. Sensory information is conveyed by projection neurons (PNs) to both calyces, where the input is further segregated into specific calyx subcompartments: the lip (LI) primarily receives information from primary olfactory brain centers, the collar (CO) primarily receives visual input from the optic lobes consisting of lamina (LA), medulla (ME), and lobula (LO), while the basal ring (BR) receives both olfactory and visual input. **b** Schematic drawing of an individual MG. Each MG comprises a single presynaptic axonal PN bouton (encircled with thick black line), which forms contacts to numerous postsynaptic profiles (PP). A large proportion of these profiles is formed by MB intrinsic Kenyon cells, but MG are also targeted by MB extrinsic neurons, such as GABAergic, dopaminergic, or octopaminergic modulatory neurons. The illustration is based on an electron microscopic image (unpublished data from Groh et al. 2012). Further abbreviations: *AL* antennal lobe, *AZ* active zone, *CX* central complex, *MT* mitochondrion, *SV* synaptic vesicle

Honeybee (*Apis mellifera*) workers exhibit a remarkable behavioral plasticity throughout their adult life. While young workers perform nursing tasks inside the dark hive, mostly guided by olfactory stimuli (e.g., pheromones), bees at an advanced age leave the hive to forage for nectar, pollen, and water. This marked transition is associated with a significant change in the sensory environment, new navigational challenges, and high cognitive demands. The behavioral plasticity is reflected by modifications at the neuronal level, particularly in the mushroom bodies (MBs; for a review see: Groh and Rössler 2020; Fig. 1a). The MBs are higher-order brain centers involved in sensory integration, spatial orientation, learning, and memory formation (e.g., Heisenberg 1998; Menzel 1999; Fahrbach 2006; Giurfa 2007, 2013). Sensory information (mainly olfactory and visual) is conveyed to the MB calyces by projection neurons (PNs), with the PN axons terminating in large presynaptic boutons that form synaptic microcircuits (microglomeruli, MG; e.g., Groh and Rössler 2020). Each MG comprises a central presynaptic PN bouton targeted by numerous postsynaptic profiles, mostly MB intrinsic neurons, so called Kenyon cells (KCs), but also by γ-aminobutyric acid (GABA)-ergic feedback neurons, and dopaminergic and octopaminergic neurons (Hammer 1993; Blenau et al. 1999; Ganeshina and Menzel 2001; Frambach et al. 2004; Fig. 1b). Based on the modality of sensory information conveyed via PNs, the MB calyces can be subdivided into distinct compartments: the lip primarily receives olfactory input from the antennal lobes, the collar visual input from the optic lobes, and the basal ring from both sensory modalities (Mobbs 1982; Gronenberg 2001; Strausfeld 2002; Fahrbach 2006; Fig. 1a).

As a response to both intrinsically and extrinsically triggered changes in the bees’ life, MG undergo significant structural reorganizations (for a review see: Groh and Rössler 2020). While increasing age and experience are associated with a general increase in MB calyx volume, mainly due to KC dendritic outgrowth, simultaneous pruning of PN boutons is promoted by non-associative sensory exposure (Withers et al. 1993; Durst et al. 1994; Fahrbach et al. 1998; Scholl et al. 2014; Muenz et al. 2015). Conversely, associative learning and stable long-term memory formation lead to an increase in PN bouton numbers (Hourcade et al. 2010; Falibene et al. 2015; Cabirol et al. 2017). Studies in *Drosophila melanogaster* recently confirmed that those PNs that had undergone associative learning contributed new PN boutons (Baltruschat et al. 2021).

The previous results suggest that behavioral plasticity in the honeybee is associated with a substantial rewiring of MG synaptic microcircuits in the MB calyces. Electron microscopy (EM) studies have provided important insights into the subcellular architecture of MG (Ganeshina and Menzel 2001; Groh et al. 2012). For instance, increasing age was shown to be associated with an increase in both the membrane surface area of visual and olfactory PN boutons, the number of active zones (AZs) per bouton, and the number of postsynaptic partners per AZ, suggesting a higher synaptic divergence of MG in aged bees (Groh et al. 2012). However, many aspects are still unanswered, for example, how exactly the synaptic microstructures change with different aspects of sensory experience.

Antibody-based immunolabeling combined with confocal laser scanning microscopy provides a valuable tool to approach these questions, but the diffraction limit of conventional light microscopy systems restricts detailed MG analyses. Expansion microscopy (ExM) can overcome this critical limitation (Chen et al. 2015). ExM is based on an isotropic physical expansion of tissue by locking proteins and immunolabels to a matrix of swellable polymers achieving up to 4.5 × expansion rates of tissues. Molecular de-crowding by ExM yields effective lateral resolution of down to ~ 60–70 nm (Chen et al. 2015; for reviews, see Gao et al. 2017; Wassie et al. 2019), which can be even further enhanced by combining ExM with super-resolution techniques such as structured illumination microscopy (SIM; Wang et al. 2018).

Since its introduction in 2015, ExM emerged to be an essential component for solving neuroscientific hypotheses and has been successfully applied for a variety of structures, tissue types, and species (e.g., RNA in mouse brains: Chen et al. 2016; human breast and kidney tissue: Zhao et al. 2017; whole *D. melanogaster* larvae: Jiang et al. 2018; sporidia of fungi: Götz et al. 2020; whole *C. elegans* preparations: Yu et al. 2020; mitochondria in human HeLa cells: Kunz et al. 2020). Furthermore, several variants of the original ExM protocol have emerged with improvements or specific applications. Among those are ultrastructural ExM (Gambarotto et al. 2021), iterative ExM (Chang et al. 2017), click-labeling ExM (Sun et al. 2021), expansion single-molecule localization microscopy (Zwettler et al. 2020), and expansion stimulated emission depletion microscopy (Gao et al. 2018). One crucial development was the introduction of protein-retention ExM (proExM; Tillberg et al. 2016). Instead of requiring custom-designed antibodies as in the original ExM protocol, proExM works with most conventional antibodies and/or fluorescent proteins by using the universal linking agent Acryloyl-X SE (AcX) to modify amines at proteins allowing them to be linked to the polymer matrix (for a review see: Wassie et al. 2019). Using this method, we applied ExM to a social insect for the first time, particularly the brain of the honeybee *Apis mellifera*. We demonstrate specific applications of the method that can be targeted at unraveling age- and experience-related structural plasticity of MB microcircuits.

## Methods

For all experiments, honeybee (*Apis mellifera*) workers were obtained from colonies at the institutional apiary at the Biocenter, University of Würzburg (Germany).

### Dissection and immunohistochemistry

#### Presynaptic proteins

To analyze age- or experience-related plasticity at MG, presynaptic AZs can serve as a proxy for changes in synaptic divergence. Therefore, we performed immunostainings against the synaptic vesicle-associated protein synapsin and the AZ-related protein Bruchpilot (BRP) in worker bees at two ages. We collected freshly emerged (≤ 24 h old) and pollen-foraging (> 3 weeks old) honeybee workers from the same colony, immobilized them on ice, and mounted the heads in dental wax-coated dishes (Modelling wax, Dentsply International, York, PA, USA) for brain dissection. Heads were covered with physiological saline (130 mM NaCl, 5 mM KCl, 4 mM MgCl_2_, 5 mM CaCl_2_, 15 mM Hepes, 25 mM glucose, 160 mM sucrose; pH 7.2), and a window was cut in the cuticle between the compound eyes, the antennal base, and the ocelli to dissect the brain. Brains were immediately fixed in ice-cold 4% formaldehyde (FA; methanol free, 28908, Fischer Scientific, Schwerte, Germany) in phosphate-buffered saline solution (PBS; 137 mM NaCl, 2.7 mM KCl, 8 mM Na_2_HPO_4_, 1.4 mM KH_2_PO_4_; pH 7.2) and incubated at 4 °C overnight. After washing in PBS and removing remaining glandular tissue, we embedded the brains in 5% low melting point agarose (Agarose II, no. 210–815, Amresco, Solon, OH, USA) and sectioned them at 80 µm using a vibrating microtome (VT 1000S, Leica Biosystems, Nussloch, Germany). We permeabilized the tissue by washing the brain sections in PBS with 2% Triton-X 100 (PBST; AppliChem, Darmstadt, Germany; 1 × 10 min) and with 0.2% PBST (2 × 10 min) and blocked unspecific binding sites by preincubation in 0.2% PBST with 2% normal goat serum (NGS; RRID:AB_2336990, Cat.No. 005–000-121, Jackson ImmunoResearch Laboratories Inc., West Grove, PA, USA) for 1 h at room temperature. We used two different primary antibodies raised against presynaptic proteins: a monoclonal mouse antibody against the *Drosophila melanogaster* synaptic vesicle-associated protein synapsin (3c11; RRID: AB_528479, kindly provided by E. Buchner, University of Würzburg, Germany; Klagges et al. 1996) and a polyclonal rabbit antibody raised against the last 200 amino acids of the *D. melanogaster* AZ-related protein BRP (BRP^last200^; kindly provided by Stephan Sigrist, FU Berlin, Germany; Ullrich et al. 2015; Gehring et al. 2017). We incubated the brain sections with both antibodies (3c11: 1:50; BRP: 1:500) in 0.2% PBST and 2% NGS for 2 days at 4 °C followed by incubation in the secondary antibodies (CF488A goat anti-mouse; 1:250; RRID:AB_10557263, Cat.No. 20018, Biotium, Fremont, CA, USA, and Alexa Fluor 568 goat anti-rabbit; 1:250; RRID:AB_143157, Cat.No. A-11011, Invitrogen by Thermo Fisher Scientific, Waltham, MA, USA) in PBS with 1% NGS for 2 days at 4 °C. After subsequent rinsing in PBS (2 × 10 min), we labeled the sections with the nuclear marker Hoechst 34580 (1:1000; Cat. No. H21486, Invitrogen by Thermo Fisher Scientific, Waltham, MA, USA) in PBS (1 × 15 min) and rinsed again in PBS (4 × 10 min). Nuclear staining was used for image calibration using KC nuclear diameters.

For further processing with ExM, we selected two agarose sections for each brain. From these sections, the agarose layer was carefully removed from the brain tissue. These sections were further processed as described in the “Expansion microscopy” section. All remaining sections were transferred to 60% glycerol in PBS and mounted on glass slides in 80% glycerol in PBS.

#### GABAergic feedback neurons

To study potential colocalization of GABA and synapsin in GABAergic feedback neurons, we double-labeled brains using antibodies against GABA and synapsin. We dissected the brains of honeybee workers of unknown age (for general procedure see previous paragraph) and immediately fixed them in ice-cold fixative solution (24.75% glutaraldehyde, 74.25% picric acid, 1% acetic acid) overnight at 4 °C. The brains were washed in PBS (5 × 20 min) and excess tissue was removed. After agarose embedding and sectioning, the sections were fixed again in 4% FA in PBS overnight at 4 °C. Tissue was permeabilized by incubation in 2% PBST (1 × 10 min) and 0.2% PBST (2 × 10 min) and blocked in 0.2% PBST with 5% NGS for 1–3 h at room temperature. For primary antibody labeling, we incubated the sections with a polyclonal rabbit antibody against GABA (1:1000; RRID:AB_477652, Cat. No. A2052, Sigma-Aldrich, St. Louis, MO, USA) and anti-synapsin (1:50) in 0.2% PBST and 2% NGS for 4–5 days at 4 °C. Then, we incubated the sections with the secondary antibodies (Alexa Fluor 568 goat anti-rabbit; 1:250 and CF633 goat anti-mouse; 1:250; RRID:AB_10582886, Cat. No. 20121, Biotium, Fremont, CA, USA) in PBS with 1% NGS for 2 days at 4 °C. After rinsing in PBS (5 × 20 min), we labeled the sections with Hoechst 34580 and proceeded analogously to the chapter presynaptic proteins.

#### Neuronal tracings combined with antibody labeling

For neuronal tracing, either freshly emerged or pollen-foraging worker bees were placed in custom acrylic holders and the head and antennae were fixed with dental wax. Then, we cut a window in the cuticle between the compound eyes, antennae, and ocelli, covered the brain in ice-cold physiological saline, and removed glandular tissue and neurolemma. For dye injection, we either used tetramethylrhodamine conjugated to biotin (3000 MW, lysine‐fixable; Micro‐Ruby; Cat. No. D7162, Invitrogen by Thermo Fisher Scientific, Waltham, MA, USA) or biotin-dextran (3000 MW, lysine fixable; DA-3000; Cat. No. D7135, Invitrogen by Thermo Fisher Scientific, Waltham, MA, USA). Crystals of both dyes were dissolved in distilled water and stored in the freezer. We then used the fine broken tip of a pulled glass capillary (KBF 112090, original diameter: 1.2/0.90 mm, Heinz Albrecht Instrumente GmbH & Co., München, Germany) to scrape some of the semi-frozen dye and injected the tip either into the antennal lobe or the MB vertical lobe. In order to trace all efferent fibers from the antennal lobe, we loaded the entire tip with dye and injected multiple times at different positions within the antennal lobe. For selective retrograde KC tracing, however, we aimed to trace only a small subset of cells and thus used as little dye as possible, barely covering the end of the glass tip. After dye application, we rinsed the brains with physiological saline, closed the head capsule and kept the bees in a dark, humid chamber for 3–4 h to let the dye diffuse. After dissection, brains were fixated in 4% FA in PBS overnight at 4 °C. All the following steps were done as described above. We complemented the neuronal tracings with an anti-synapsin labeling (1:50; secondary antibody: CF633 goat anti-mouse, 1:250), and some of them also with an anti-BRP labeling (1:500; secondary antibody: Alexa Fluor 568 goat anti-rabbit, 1:250). Furthermore, we post-labeled neuronal tracers with streptavidin for signal amplification (1:250; either streptavidin Alexa Fluor 488 conjugate, Cat. No. S11223, or streptavidin Alexa Fluor 568 conjugate, Cat. No. S11226, Invitrogen by Thermo Fisher Scientific, Waltham, MA, USA) in PBS with 1% NGS overnight at 4 °C, and labeling with Hoechst 34580 (1:1000) was added as described above.

### Expansion microscopy

For tissue expansion, we followed the basic proExM protocol for intact tissues by Asano et al. (2018). In order to perform the anchoring of biomolecules and immunolabels, we resuspended Acryloyl-X SE (AcX; 6-((acryloyl)amino)hexanoic acid, succinimidyl ester, Cat. No. A20770, Invitrogen by Thermo Fisher Scientific, Waltham, MA, USA) in anhydrous dimethyl sulfoxide (≥ 99.9%; DMSO; Cat. No. 276855, Sigma-Aldrich, St. Louis, MO, USA) at 10 mg/ml and incubated the brain sections in AcX/DMSO in PBS (1:100) overnight at room temperature. After washing the sections in PBS (3 × 15 min), we prepared the gelling solution on ice by mixing monomer solution (for recipe see: Asano et al. 2018), 4-hydroxy-TEMPO (4HT; Cat. No. 176141, Sigma-Aldrich, St. Louis, MO, USA), N,N,N′,N′-Tetramethylethylenediamine (TEMED; Cat. No. T7024, Sigma-Aldrich, St. Louis, MO, USA) and Ammonium persulfate (APS; Cat. No. A3678, Sigma-Aldrich, St. Louis, MO, USA) in a ratio of 47:1:1:1. The sections were covered with the gelling solution and incubated for 5 min at room temperature on a shaker. Afterwards, the sections were transferred into fresh gelling solution and incubated for 25 min on ice. We built custom gelation chambers by cutting coverslips (high-precision microscope cover glasses 1.5H, Cat. No. 0107222, Paul Marienfeld GmbH & Co. KG, Lauda-Königshofen, Germany) with a diamond knife and sticking the pieces to a glass slide with a droplet of water. We transferred the sections into the chamber with a brush, covered them with ~ 90 µl of gelling solution, and sealed the chamber with a coverslip. The whole gelation chambers were placed in a humid 1-well plate, sealed with parafilm and transferred into an incubator at 37 °C for 2–2.5 h for polymerization. After the gelation process, we removed the cover glass with a razor blade, trimmed the hydrogel around the brain tissue, and transferred all samples into a 6-well plate with digestion buffer (for recipe see: Asano et al. 2018) and proteinase K (8 U/ml; Cat. No. AM2546, Invitrogen by Thermo Fisher Scientific, Waltham, MA, USA) overnight at room temperature. After digestion, we added another labeling cycle with the respective primary and secondary antibodies for each experiment (for concentrations and incubation times see previous paragraphs) to increase staining quality. Finally, for tissue expansion, we transferred the gels into petri dishes and washed them with ddH_2_O (6 × 15 min). Using a coverslip, we transferred the expanded gels into self-adhesive silicone imaging chambers (CoverWell™ Imaging Chambers, Cat. No. 635011, Grace Bio-Labs, Bend, OR, USA), added a few microliters of water to the gel and placed the chambers on glass slides for imaging.

### Image acquisition and processing

All samples were scanned with a confocal laser scanning microscope (Leica TCS SP8 MP, Leica Microsystems AG, Wetzlar, Germany) using either a 20 × multi-immersion objective (HC PL APO 20x/0.75 IMM CORR CS2) or a 63 × glycerol immersion objective (HC PL APO 63x/1.30 Glyc CORR CS2) with additional digital zoom. For excitation, we either used a 488-nm OPSL, 552-nm OPSL, or 638-nm diode laser (Coherent, Santa Clara, CA, USA) and a multiphoton laser (InSight DeepSee Dual, Spectra-Physics, Santa Clara, CA, USA) for UV excitation. Depending on the signal strength, either internal photomultipliers or a hybrid detector was used for signal detection. All images were taken at a frame size of 1024 × 1024 pixels. For the quantification and 3D reconstruction of presynaptic proteins at MG, we recorded image stacks of individual MG with a z-step size of 0.33 µm. To determine the expansion factor of each preparation and calibrate individual preparations, we scanned Hoechst 34580-labeled KC nuclei in both the expanded sections and an unexpanded glycerol section of the same brain with the same magnification (20 × with a 0.75 × digital zoom).

All multi-channel microscope images were merged in the ImageJ distribution Fiji (version 1.53 k; Schindelin et al. 2012), adjusted for brightness and contrast and smoothened with the “Gaussian blur” filter and later arranged and annotated using CorelDraw 2018 (version 20.1.0.708, Corel Corporation, Ottawa, Canada). We used the landmark function of the software Amira 2019.1 (FEI, Visualization Sciences Group, Hillsboro, OR, USA) to quantify BRP clusters in the presynaptic protein image stacks and created three-dimensional reconstructions of MG with the segmentation editor and the SurfaceGen module. Barplots for the BRP quantification (Fig. 6) were created in RStudio (version 1.3.1073) with the ggplot2-based package ggpubr (version 0.4.0; Kassambara 2020). For structural comparison of our KC tracings in combination with anti-synapsin and anti-BRP immunolabeling (Fig. 8f) and as a basis for our schematic drawing of a MG (Fig. 1b), we used an electron microscopic image selected from unpublished data from Groh et al. (2012).

### Calculation of expansion factors and distortion analysis

For each sample, we determined the expansion factor by measuring the diameter of 10 inner non-compact KC nuclei with Fiji in an expanded brain section and in a different, unexpanded agarose section of the same brain.

Furthermore, we performed an exemplary distortion analysis on two selected samples to validate the isometry of tissue expansion. For this, we mounted conventional agarose brain sections labeled with the nucleic acid stain Sytox green (1:10000; Cat. No. S7020, Invitrogen by Thermo Fisher Scientific, Waltham, MA, USA) on glass slides and recorded an image stack of a whole non-compact KC nucleus at the confocal laser scanning microscope with a 63 × objective, a 4 × digital zoom, and a z-step size of 0.33 µm. Afterwards, we removed the brain sections from the glass slide and proceeded with the ExM protocol. The expanded section was then transferred to the microscope, and an image stack of the same nucleus was scanned with a 63 × objective, a 2 × digital zoom, and a z-step size of 0.33 µm.

To map pre-expansion with post-expansion images, we implemented a similarity transform using Elastix (Marstal et al. 2016). The similarity transform is composed of four operations: a shift in x and y directions, a scaling, and a rotation. Therefore, it only corrects the isotropic expansion (scaling) and the varying position under the microscope (translation x, y, and rotation). If the overlay of pre- and post-ExM images is sufficiently accurate, we assume that the optimization process succeeded and accept the scaling factor of the similarity transform as structural expansion factor. Pearson correlation index was computed to evaluate the degree of overlap of pre- and post-ExM images (Adler and Parmryd 2010). Following this step, we further determined nonlinearities in the expansion processes by computing a B-spline transform of the similarity transformed pre-expansion image. The B-spline transform corrects the remaining nonlinearities and, therefore, gives an estimation of the occurring distortions. Transforming mesh grids with the size of the similarity transformed image yields the corresponding vectorial shift. We draw these vectors in the similarity transformed image to create our distortion maps. This custom script implementing the workflow in python has been described and applied to ExM data before (Trinks et al. 2021).

## Results

**Fig. 2:**
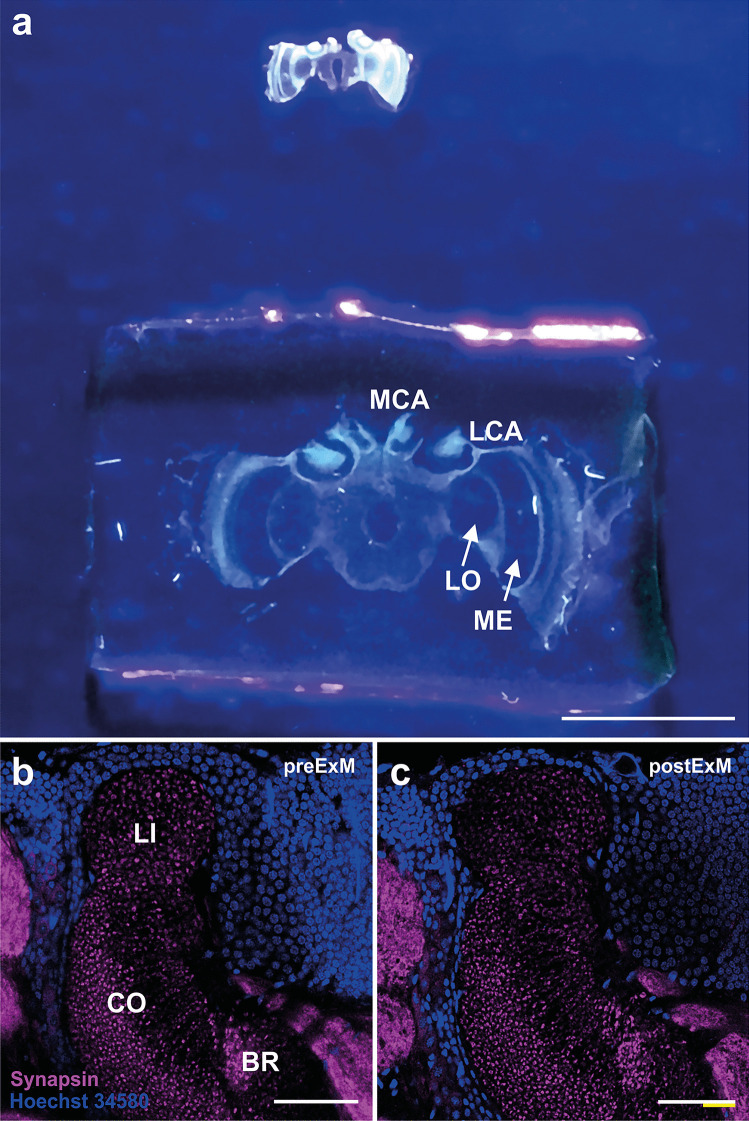
Pre- and post-expansion comparison of honeybee brain structures.** a** Comparison of an agarose-embedded brain section of a honeybee worker (upper) and the expanded brain section embedded in the hydrogel (lower). Both sections were labeled with the nuclear marker Hoechst 34580 and imaged under UV light. The tissue size has linearly increased by a factor of ~ 3. Due to the increased size, major neuropils can be distinguished without magnification: the medial (MCA) and lateral (LCA) mushroom body (MB) calyces, the lobula (LO) and the medulla (ME) of the optic lobes. **b** Synapsin-immunostained MB calyx (magenta) with the three subcompartments lip (LI), collar (CO) and basal ring (BR), and Hoechst-34580 stained Kenyon-cell (KC) nuclei (blue) prior to expansion. **c** The same MB calyx as in **b** after expansion. Tissue has linearly expanded by a factor of ~ 2.4. Scale bars: 5 mm (**a**), 50 µm (**b, c**). White bar: biological size before expansion, yellow bar: physical size after expansion

**Fig. 3:**
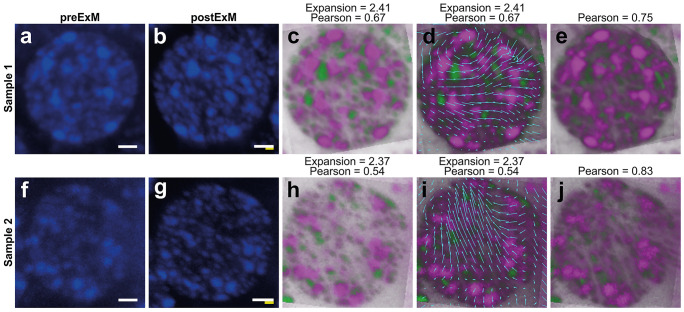
Distortion analysis of Kenyon cell (KC) nuclei using Elastix. To determine an estimated expansion factor of pre-expansion (**a, f**) and post-expansion (**b**, **g**) images and a vector map that describes the distortions occurring during the expansion process, we use the processing pipeline SimpleElastix (Marstal et al. 2016) as described in Trinks et al. (2021). First, the pre-expansion image (green) is aligned with a similarity transform with the post-expansion image (magenta) (**c**, **h**). In a second step, the pre-aligned image is further registered with a B-spline transform (**e**, **j**). The B-spline transform yields a smoother alignment, but also includes nonlinearities. By extracting the translation vector of the transformation’s underlying knots, the occurring distortions can be estimated, since these represent the differences between the best possible linear and the “perfect” nonlinear alignment (**d**, **i**). Scale bars: 1 µm (**a**, **b**, **f, g**). White bar: biological size before expansion. Yellow bar: physical size after expansion

To investigate ultrastructural details of MB synaptic complexes in the honeybee with light microscopy, we used proExM in combinations with different antibody labeling and neuronal tracing. We successfully expanded honeybee brains with expansion factors ranging between 2.3 and 4.5 (mean = 3.34 ± 0.65 (s.d.); Fig. 2a). Using the proExM protocol, the structural integrity of the tissue was preserved indicating nearly isometric expansion (Fig. 2b, c), which was confirmed by exemplary subsequent distortion analyses of pre- and post-expansion images of non-compact KC nuclei (Fig. 3).

For the two samples analyzed, these analyses revealed expansion factors of 2.41 and 2.37, respectively. By using Elastix (Marstal et al. 2016), we computed a similarity transform (Fig. 3c, h) which maps pre- with post-expansion images and allows to generate distortion maps (Fig. 3d, i). These analyses yielded Pearson correlation coefficients of 0.75 and 0.83 which are in line with earlier ExM data from Trinks et al. (2021).

Fluorescence signal strength in expanded specimen was generally lower than in conventional samples, but by adding a second antibody labeling cycle, the signal strength could be substantially improved. For this, we decided to use post-digestion labeling rather than post-expansion labeling, as we did not find any substantial differences in signal strength between both methods.

### Presynaptic proteins

AZs in presynaptic PN boutons are one potential proxy to estimate sites of synaptic transmission in individual MG. It is, therefore, a crucial asset to visualize AZs at the light microscopic level as it has been done in *D. melanogaster* using STED microscopy (Kittel et al. 2006). In the honeybee, a first light microscopy-based attempt to visualize AZ-related structures at MG by Gehring et al. (2017) provided promising insights, but the diffraction barrier put a limitation to deeper investigation. Thus, detailed characterization of AZs has so far only been achieved by time-consuming serial-section EM. Using an antibody that targets the *D. melanogaster* AZ-related protein BRP (Ullrich et al. 2015) and combining it with ExM, we aimed to overcome this limitation and to assess changes in numbers of AZ-related BRP clusters at presynaptic PN boutons in young nurse bees in comparison with old forager bees. Furthermore, we aimed to resolve localization of BRP and the synaptic vesicle-associated protein synapsin within axonal PN boutons.

**Fig. 4:**
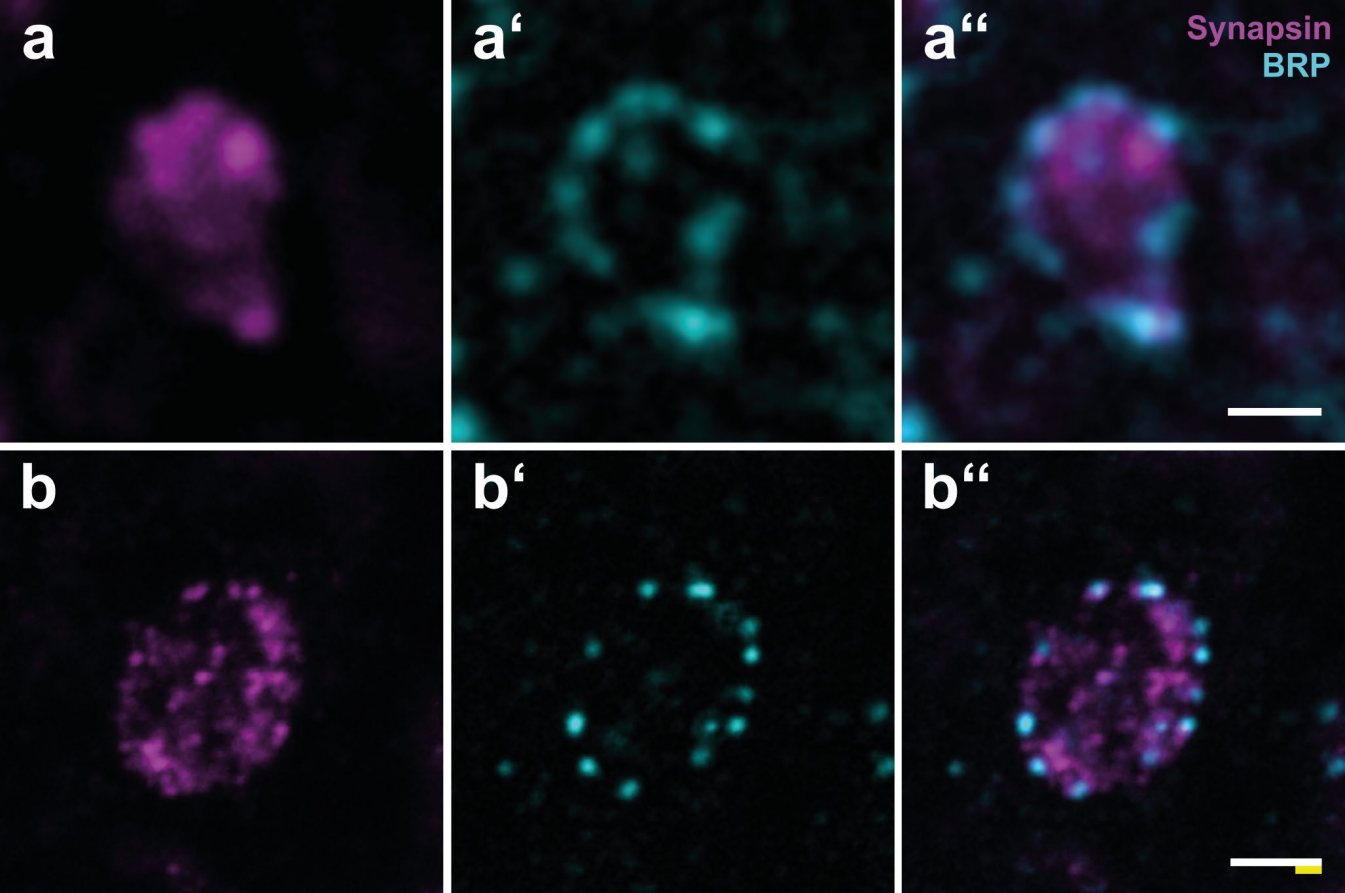
Pre- and post-expansion comparison of projection neuron (PN) boutons double-immunolabeled against synapsin and Bruchpilot (BRP). **a-a’’** Synapsin-immunostaining (magenta, **a**), BRP-immunostaining (cyan, **a’**) and the overlay of both signals (**a’’**) in a single PN bouton in the dense collar of the mushroom body (MB) calyx in an agarose section without expansion microscopy (ExM). Synapsin-immunostained synaptic vesicles are arranged as a coherent structure which is surrounded by several large clusters of BRP. **b-b’’** Synapsin-immunostaining (**b**), BRP-immunostaining (**b’**), and the overlay of both signals (**b’’**) in a single PN bouton in the dense collar of the MB calyx after ExM. PN boutons in **a** and **b** have been chosen for illustration purposes only and are not from the same preparation. Scale bars: 1 µm. White bar: biological size before expansion. Yellow bar: physical size after expansion

**Fig. 5:**
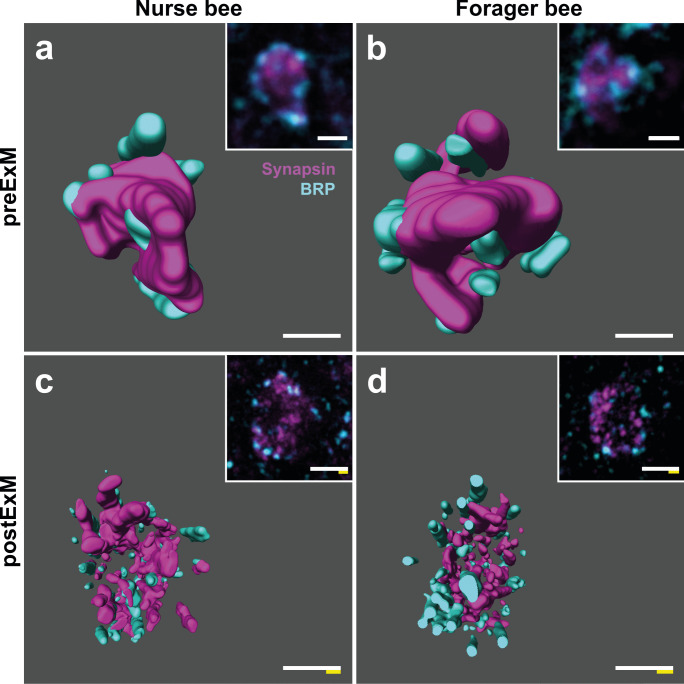
3D reconstructions of projection neuron (PN) boutons double-immunolabeled against synapsin and Bruchpilot (BRP) in nurse and forager bees with and without expansion microscopy (ExM). BRP signals (cyan) are arranged alongside the outer edges of synapsin-positive (magenta) PN boutons. Young nurse bees (**a**, **c**) exhibit a lower number of BRP-immunoreactive clusters than old forager bees (**b**, **d**). Without ExM (**a**, **b**), BRP clusters appear as large coherent clusters, while preparations processed with ExM (**c**, **d**) reveal that these clusters consist of many small substructures which allow for a detailed quantification of individual spots. Insets in **a**–**d** show corresponding microscope images for better visualization. Scale bars: 1 µm. White bar: biological size before expansion. Yellow bar: physical size after expansion

Both synaptic antibodies yielded a good pre- and post-expansion immunostaining quality, and the spatial resolution of both antibody signals was significantly increased (Fig. 4). While in unexpanded tissue, synapsin-immunoreactive (IR) clusters appeared as a coherent structure surrounded by blurry BRP-IR clusters (Fig. 4a’’ and Fig. 5a, b), with ExM we were able to resolve both antibody clusters as distinct, clearly delimited clusters of approximately the same size (Fig. 4b’’ and Fig. 5c, d).

The average xy-diameter of a single BRP assembly decreased from 485 nm in unexpanded preparations to only 122 nm (after correction for tissue expansion) in expanded samples emphasizing the substantial gain of spatial resolution. While synapsin-IR was densely clustered throughout the entire PN bouton, BRP signals were usually restricted to the outer rim of presynaptic boutons classified by anti-synapsin labeling. Notably, BRP clusters were also found outside of designated PN boutons (Fig. 4), but we restricted our quantitative analyses to synapsin-positive MG.

Exemplary analyses included samples of 5 expanded brains of honeybees (3 nurse bees and 2 forager bees) with an average expansion factor of 3.78 (± 0.24 (s.d.)).

**Fig. 6:**
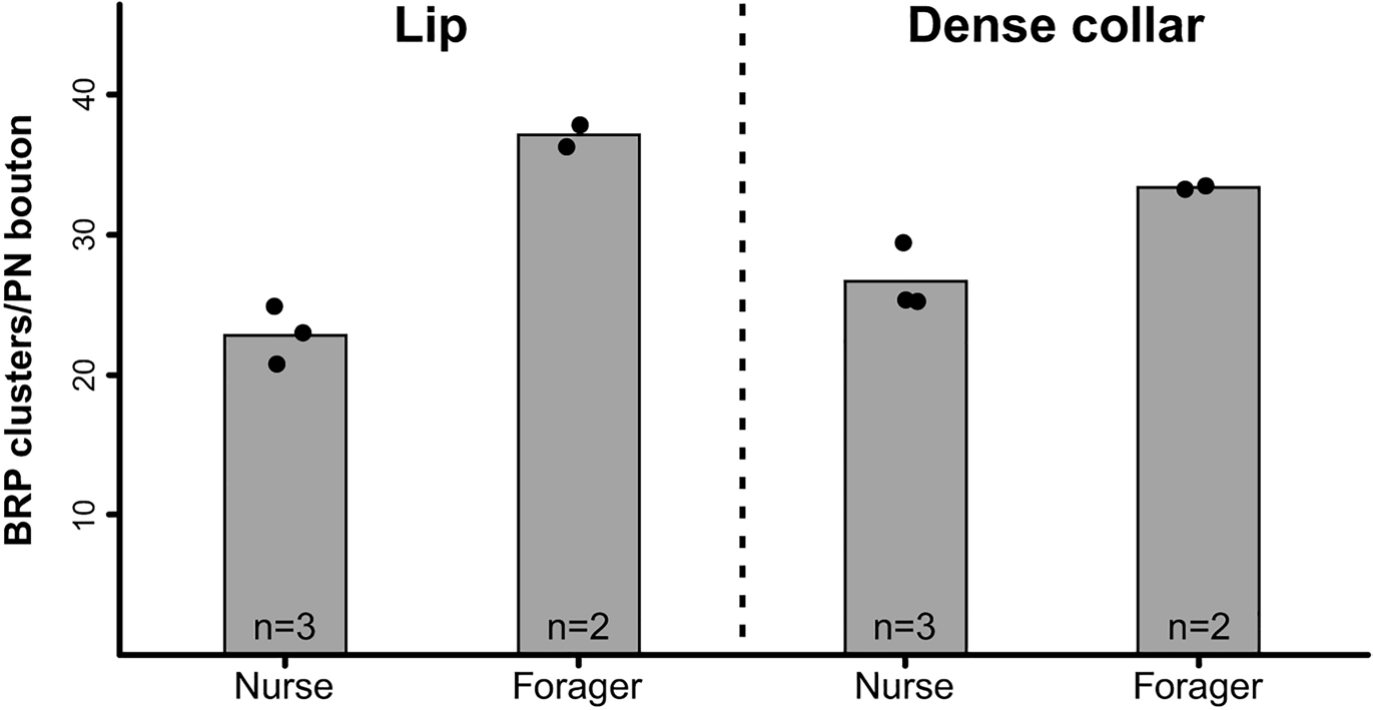
Exemplary Bruchpilot (BRP) quantification at projection neuron (PN) boutons in the mushroom body (MB) calyx of nurse and forager bees. Left: Mean number of BRP clusters per PN bouton in the MB lip of nurse and forager bees based on three-dimensional quantifications. In total, 19 PN boutons were analyzed. Right: Mean number of BRP clusters per PN bouton in the MB dense collar of nurse and forager bees based on three-dimensional quantifications. In total, 24 PN boutons were analyzed. Barplots represent the mean of each group, single dots represent the mean over all analyzed PN boutons of the same animal

We quantified BRP-IR clusters in a total of 43 MG in olfactory and visual subdivisions of the MB calyces (19 MG in the lip, 24 MG in the dense collar). The analysis revealed that forager bees had ~ 40% more BRP clusters per MG than nurse bees (nurses: 24.73 ± 2.89 (s.d.); foragers: 35.26 ± 2.31 (s.d.)), while numbers of both age groups appeared similar in the lip and dense collar region (lip: 28.56 ± 8.01 (s.d.); dense collar: 29.33 ± 4.04 (s.d.); Figs. 5 and 6).

### GABAergic feedback neurons

**Fig. 7:**
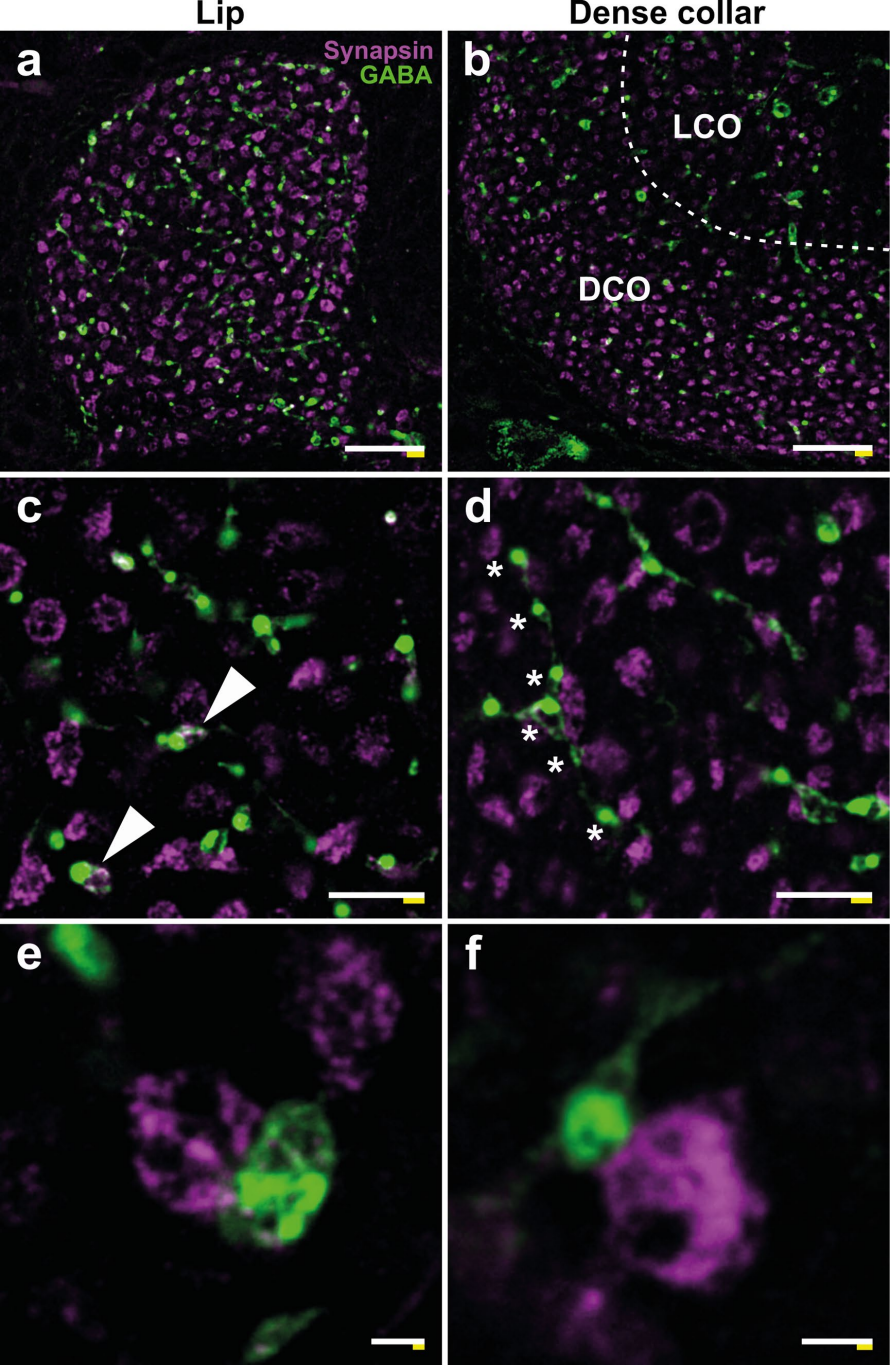
GABAergic innervation in lip and dense collar of the mushroom body (MB) calyx. **a**, **b** Overviews of synapsin (magenta)- and GABA (green)-positive profiles in the lip (**a**) and dense collar (**b**) of the MB calyx. The dashed line marks the boundary between dense (DCO) and loose collar (LCO). **c**, **d** Detailed views of the distribution of anti-synapsin- and anti-GABA-immunoreactivity (IR) in the lip (**c**) and dense collar (**d**). Most of the GABAergic profiles form direct contacts to synapsin-positive projection neuron (PN) boutons, but sometimes also enwrap them as a ring-like structure (arrowheads in **c**). Axonal boutons often appear in a thread-like arrangement (asterisks in **d**) contacting several neighboring PN boutons. **e** High-resolution image of a large GABAergic bouton contacting a PN bouton in the lip region. **f** High-resolution image of a small GABAergic bouton contacting a PN bouton in the dense collar region. No overlap of both markers is discernible. Scale bars: 10 µm (**a**, **b**), 3 µm (**c**, **d**), 500 nm (**e**, **f**). White bar: biological size before expansion. Yellow bar: physical size after expansion

GABAergic feedback neurons are MB output neurons that connect the MB lobes with the MB calyces (Schäfer and Bicker 1986; Rybak and Menzel 1993; Grünewald 1999a), where they arborize and form synaptic contacts with both PN boutons and KCs (Ganeshina and Menzel 2001). Quantifications of MG within different calyx compartments have been frequently performed using detection of synapsin-IR presynaptic PN boutons (discussed in Fahrbach and Van Nest 2016). Whether large profiles of GABAergic feedback neurons contribute to these numbers remained unclear. To address this question and to further dissect the microarchitecture of pre- and postsynaptic partners within MG, we performed double-immunolabeling against synapsin and GABA and high-resolution confocal imaging using ExM (Fig. 7).

The anti-GABA antibody penetrated the brain sections 10–20 µm and yielded excellent staining qualities within this range that persisted throughout the expansion process (Fig. 7). GABAergic profiles were present in both the lip and dense collar, but the GABAergic network was more densely distributed in the olfactory lip region (Fig. 7a, b). Usually, GABAergic boutons were found in proximity or direct contact with synapsin-IR PN boutons (Fig. 7c, d). Like results from a previous EM study (Ganeshina and Menzel 2001), we observed small GABA boutons with a xy-diameter of ~ 500 nm (Fig. 7f) comprising the majority of GABAergic boutons in the MB calyx. Occasional large GABA boutons with a xy-diameter of ~ 1–1.5 µm exhibited a similar size as PN boutons (Fig. 7e). With the increased resolution of ExM, we were able to identify differences in the relative arrangement of GABAergic boutons and PN boutons: small GABA boutons frequently appeared as solid structures arranged in a thread-like sequence contacting several neighboring PN boutons (Fig. 7d, asterisks). Close proximities between both bouton types were on the surface and restricted to small contact areas (Fig. 7e). However, we also observed isolated ring-like GABA boutons, sometimes encircling the entire PN bouton (Fig. 7c, white arrowheads) or an intermingled arrangement of GABA boutons and PN boutons (Fig. 7e). Most interestingly, we never found overlap or colocalization of anti-synapsin- and anti-GABA-IR within the same structure indicating that the boutons of GABAergic feedback neurons are devoid of synapsin.

### Expansion of neuronal tracings combined with antibody labeling

**Fig. 8:**
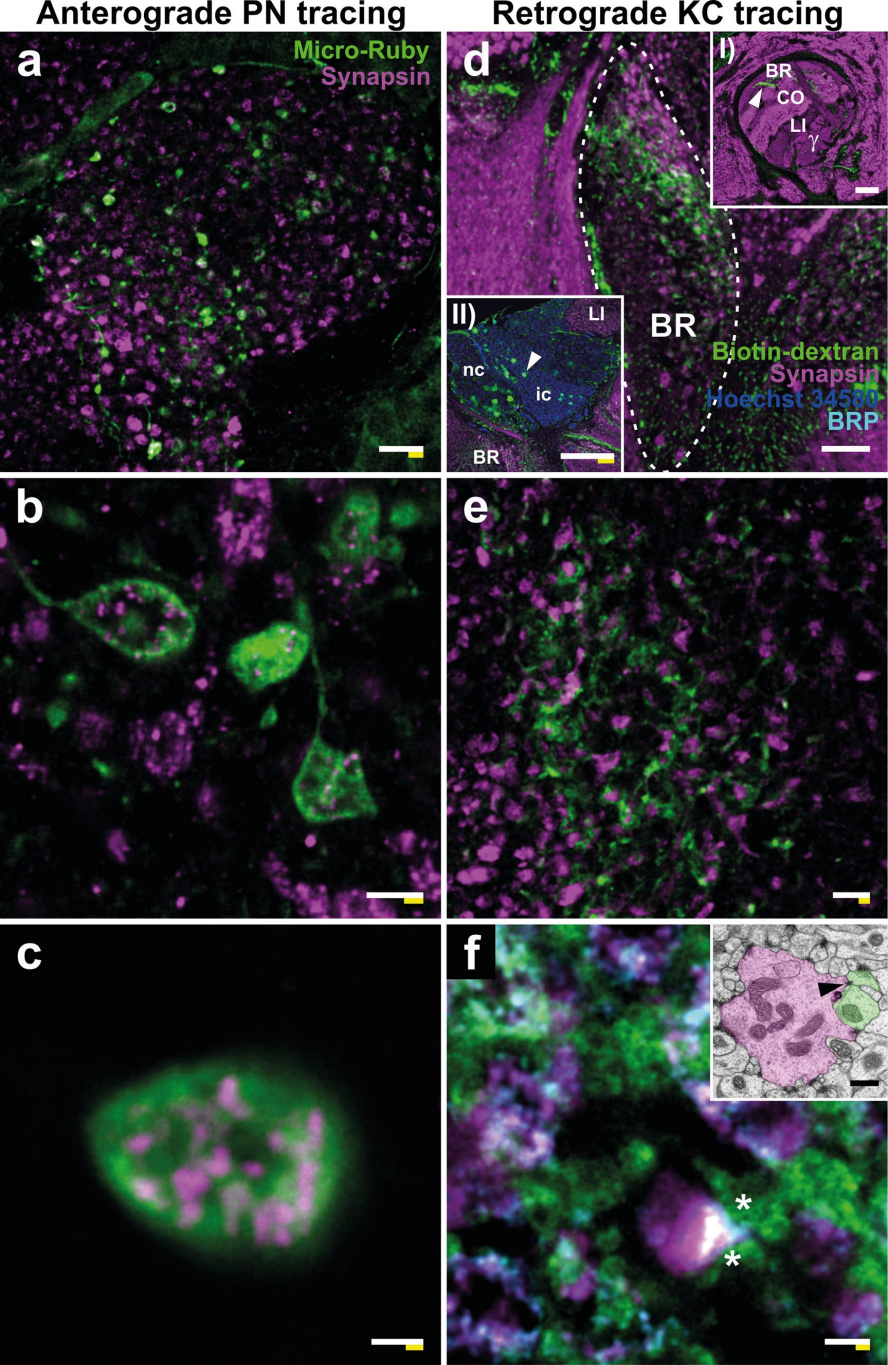
Neuronal tracings of projection neurons (PNs) and mushroom body (MB) Kenyon cells (KCs).** a–c** Anterograde tracing of antennal lobe (AL) PNs. **a** An expanded MB lip immunolabeled against synapsin (magenta) showing PN boutons (green) labeled via dye injection into the AL. **b** Detailed view of PN boutons and anti-synapsin-immunoreactivity (IR) from the MB lip. **c** High magnification of a single PN bouton in the basal ring (BR). **d–f** Retrograde tracings of class I KCs from the vertical lobe. **d** Overview of the KC tracings in the BR (encircled by dashed line). Inset I: MB vertical lobe. The point of dye injection is indicated by the white arrowhead. The labels depict the layering of KC axon terminals from the basal ring (BR), the collar (CO) and the lip (LI), and the axon terminals of class II KCs (γ lobe). Inset II: Overview of KC nuclei showing the respective nuclei of KCs labeled via dye injection (green). The arrowhead points to a labeled KC nucleus within the cluster of class I inner compact cells (ic). **e** Detailed view of labeled KC dendrites (green) in the BR. The anti-synapsin labeling (magenta) depicts presynaptic vesicles at PN boutons. **f)** High magnification view of KC dendrites (green), PN boutons (magenta), and Bruchpilot (BRP) clusters (cyan). Asterisks indicate putative synaptic contacts. Inset: Electron microscopy image of a PN bouton (magenta) in the lip of a freshly-emerged bee (unpublished data from Groh et al. 2012). The arrowhead indicates a ribbon synapse contacting two postsynaptic profiles (green). Further abbreviations: *nc* non-compact cells. Scale bars: 10 µm (**a**), 2 µm (**b**), 500 nm (**c**, inset in** f**), 20 µm (**d**, inset II in** d**), 50 µm (inset I in** d**), 5 µm (**e**), 1 µm (**f**). White bar: biological size before expansion. Yellow bar: physical size after expansion

In addition to antibody immunostainings, neuronal tracings are frequently used to characterize the connectivity of neuronal circuits. However, to our knowledge, up to now neuronal tracings via dye injection have never been processed with ExM. We successfully combined both neuronal tracings and antibody staining with ExM in the honeybee brain. To visualize pre- and postsynaptic elements of MG, we used anterograde injection into the antennal lobe to label PNs ascending into the MB calyx lip and basal ring (Fig. 8a–c). In addition, retrograde labeling of KC dendrites was achieved by dye injection into the MB vertical lobe (Fig. 8d, inset I and Fig. 8d–f). We found that biotinylated dextrans are suited for neuronal tracing and detection by subsequent ExM. However, post-labeling with streptavidin was required to enhance labeling due to a significant signal loss. We were able to label presynaptic PN boutons in both the MB lip (Fig. 8a, b) and basal ring (Fig. 8c).

In contrast to synapsin antibody labeling, neuronal tracing of PNs allowed us to reveal that olfactory PN boutons exhibit different shapes, ranging from spheres to very thin ring-like structures (Fig. 8b). They are usually accompanied by an overlapping cluster of synapsin-positive structures (Fig. 8c). However, we observed substantial variation in the distribution and amount of synapsin-IR clusters across different PN boutons (Fig. 8b, c).

By inserting dye into the basal ring region of the vertical lobe, we successfully stained a subset of MB KCs (Fig. 8d). Based on the point of dye injection (Fig. 8d, inset I) and the location of labeled KC somata (Fig. 8d, inset II), the traced neurons can be classified as class I (spiny) KCs (Groh and Rössler 2020). They arborized in a dense web around synapsin-labeled presynaptic MG sites in the basal ring (Fig. 8d, e). Due to the dense ramifications, it was not possible to identify and track individual neurons to quantify the number of synaptic contacts. However, by combining presynaptic synapsin labeling and postsynaptic KC tracing with the AZ-marker BRP, the microarchitecture of MG could be visualized in remarkable detail (Fig. 8f, see comparison with EM image). Postsynaptic KC profiles occupy a large proportion of the presynaptic bouton area. However, only a limited part of these potential contact areas is associated with an accumulation of BRP, potentially indicating sites of active synaptic transmission (Fig. 8f).

## Discussion

We successfully applied ExM for the first time in a social insect species, the western honeybee *Apis mellifera*. Our study demonstrates that this method can readily be used in various combinations of established immunohistochemical and neuronal tracing protocols providing a valuable baseline for other social insect species. This approach yields improved insights into the microstructure of MG in the honeybee MBs at the light microscopic level. By reaching expansion factors of up to 4.5, we overcame the diffraction limit of conventional light microscopic systems and were able to localize and visualize presynaptic epitopes at a new level of detail and further broaden this approach by co-labeling of postsynaptically connected neurons via dye injection. We found that synapsin and BRP assemblies, two important components in the presynaptic architecture of MG, can be visualized and quantified at a subdiffraction scale and thus be used as a proxy for age-, experience-, or learning-related synaptic plasticity. Furthermore, we confirmed that synapsin-IR is absent in GABAergic profiles from a group of MB feedback neurons in the MB calyces, which opens up new perspectives for future MG circuit analyses. To our knowledge, ExM has never been used for processing of neuronal tracings before. In combination with antibody immunostaining, this allows valuable insights into the fine structure of PN boutons, dendritic specializations of KCs, and their synaptic connections.

### Presynaptic proteins

In *Drosophila melanogaster*, BRP is accumulated at the AZ, where it concentrates synaptic vesicles close to clusters of Ca^2+^ channels (Wagh et al. 2006; Fouquet et al. 2009; Kittel and Heckmann 2016). In concert with synapsin, BRP influences neurotransmitter release at synapses (Greengard et al. 1993; Wagh et al. 2006). Although the function of BRP in the honeybee is not fully understood yet, it seems likely that it serves a similar function like in *Drosophila*, rendering synapsin and BRP eligible candidates to estimate synaptic activity at MG. A study by Gehring et al. (2017) analyzed the localization of BRP in the honeybee brain for the first time using the same antibody as in the present study. They found that BRP (here AmBRP) is localized near the presynaptic membrane of PN boutons and reported an age-related increase in relative AmBRP levels. However, quantification in this study was based on intensity measurements in two-dimensional regions of interest, which is prone to variances caused by differences in staining quality. With the increased resolution of ExM, we were able to analyze the three-dimensional representation of BRP and synapsin colocalization at PN boutons and to perform age-related quantifications of clearly delimited BRP-IR clusters at individual boutons. The 3D structure revealed that BRP-IR is exclusively localized in the outer rim of PN boutons (Figs. 4 and 5), which supports a similar role of BRP in synaptic transmission like in *Drosophila*. Although our sample size does not allow detailed statistical analyses, the age-related difference in the number of BRP-IR assemblies in both calyx regions (Fig. 6) seems to hint in the same direction as the data of Gehring et al. (2017). This is also in line with results from a serial-section EM study by Groh et al. (2012), which detected an age related-increase in the number of AZs per PN bouton while the overall number of PN boutons decreases with age. This results in a net increase of synaptic divergence between PN boutons and synaptically connected KCs by over 30%. Although the trend in our ExM results confirms the study by Groh et al. (2012), we detected a lower number of BRP-IR clusters per PN bouton in comparison with the number of AZs detected via serial EM. BRP-IR clusters visualized by ExM had an average diameter of ~ 120 nm, which is in the range of an individual AZ at EM resolution (Groh et al. 2012; Fig. 8f, inset). Therefore, the resolution achieved with ExM should in principle allow us to discern single AZs. In *Drosophila*, BRP is essential for the formation of presynaptic bars at synapses, often taking the shape of so-called T-bars (Fouquet et al. 2009; for a review see: Wichmann and Sigrist 2010). Electron-dense projections are also found at synapses of honeybee PN boutons (Groh et al. 2012; Fig. 8f, inset) and other insect species (for a review see: Schürmann 2016). However, in the honeybee, AZs rarely exhibit the shape of a T-bar (pedestal with platform) as described in *Drosophila*, but mature AZs rather have a ribbon-shaped structure (only a pedestal; Groh et al. 2012). In fact, a large proportion of AZs has a non-ribbon shape, especially in very young bees (Groh et al. 2012). We therefore hypothesize that BRP may not necessarily be found at all AZs in the honeybee MB calyces, but only at those in mature ribbon synapses, which would explain a lower overall number of BRP spots compared to AZs. In the same line, this would explain the higher number of BRP-IR spots in foragers. Overall, our findings suggest that BRP is associated with AZs and synaptic transmission in the honeybee and undergoes age-related changes associated with an increase in synaptic divergence at PN boutons. To further elucidate the exact localization and potential function of BRP at honeybee MB synapses, a correlative approach of light- and electron microscopy might be the method of choice. Array tomography, for example, provides a powerful tool to combine the benefits of specific antibody immunostainings and the high ultrastructural resolving capacity of scanning EM and was already successfully used for detailed MG characterization in the honeybee and the desert ant *Cataglyphis fortis* (Markert et al. 2017).

### GABAergic feedback neurons

In each brain hemisphere of the honeybee, approximately 50 GABAergic neurons of the A3v cluster connect the MB lobes with the calyces as recurrent feedback neurons, where they exhibit an important modulatory function (Schäfer and Bicker 1986; Rybak and Menzel 1993; Grünewald 1999a, b; Haehnel and Menzel 2010; Raccuglia and Mueller 2013; Zwaka et al. 2018). In both the honeybee and *Drosophila*, these neurons function in gain control and normalizing sparse KC activity levels (Haehnel and Menzel 2012; Prisco et al. 2021). Previous structural analyses of age- and experience-related changes in the MB calyces mainly focused on quantification of PN boutons based on synapsin immunolabeling as proxy for MG density (e.g., Groh et al. 2006, 2012; Muenz et al. 2015; Cabirol et al. 2017). Due to their large size and clearly delineated spheroidal shape, they are easy to detect (discussed in Groh and Rössler 2020). However, the question was raised whether large GABAergic boutons might contribute to these quantifications, too. Earlier studies reported the presence and/or functional implications of synapsin in GABAergic synapses, e.g., in local interneurons in *Drosophila* where synapsin function is required for short-term habituation of olfactory avoidance behavior (Sadanandappa et al. 2013), in hippocampal interneurons in mice (Song and Augustine 2016; Forte et al. 2020), and even in GABAergic profiles in the honeybee MB peduncle (Okada et al. 2007). The colocalization of GABA and synapsin at synapses in the MB calyces of the honeybee, however, has not been studied in detail yet. Confirming earlier EM analyses (Ganeshina and Menzel 2001), our ExM analyses show that GABAergic boutons and synapsin-positive PN boutons in the honeybee MB calyces often are in close apposition. However, we never detected synapsin-IR within any GABAergic profiles. This represents an important finding as it shows that GABAergic boutons in the honeybee MB calyx do not label with anti-synapsin antibody and therefore do not contribute to PN bouton counts based on synapsin-IR. This also allows differential assessment of both types of boutons. The result further indicates that GABAergic feedback neurons in the honeybee MBs might have a synapsin-independent mechanism of synaptic transmission. The main role of synapsin is clustering and tethering synaptic vesicle reserve pools to the actin cytoskeleton of the presynapse and modulating their availability upon stimulation (for a review see: Cesca et al. 2010). Thus, it represents an essential component of short-term and long-term synaptic plasticity with substantial impact on learning and memory formation. As the activity of A3v GABAergic feedback neurons was shown to express changes after olfactory learning (Grünewald 1999b), this function is most likely implemented by a different mechanism or, alternatively, by a different synapsin isoform that is not detected by the antibody. Consequently, the underlying mechanisms of long-term plastic changes in the GABAergic network in the context of age-, experience-, or learning-related MB plasticity are an interesting field for future studies as well as analyzing the colocalization of synapsin and GABA at GABAergic synapses in other regions of the honeybee brain.

We found that GABAergic boutons in the lip and dense collar appear in different sizes, shapes, and spatial arrangements with PN boutons. For example, we found boutons with a size comparable to PN boutons (~ 1.5–2 µm), but also very small boutons (~ 500 nm), which correspond to the classification into principal boutons and accessory boutons in an EM study by Ganeshina and Menzel (2001). Furthermore, we found contacts between GABAergic boutons and PN boutons to be either superficial and restricted to a small contact area or distributed over a larger region, with GABAergic profiles sometimes wrapping around PN boutons. This variety among GABAergic boutons might be correlated with a functional diversity: GABAergic boutons in the MB calyces do not only form synapses with postsynaptic KCs and PN boutons, but also receive synaptic input from PN boutons, most likely forming local inhibitory circuits (Ganeshina and Menzel 2001). In this context, it would be highly interesting to combine our approach with AZ markers like BRP to identify active boutons and exact sites of synaptic transmission. Furthermore, we observed that thread-like axonal GABAergic boutons along the same neurite form contacts with several PN boutons. It is unknown whether these groups of PN boutons belong to a functionally similar subpopulation (e.g., activated by the same odorant) or whether one GABAergic neuron may exhibit its modulatory function across functionally different populations of PNs.

### Neuronal tracings combined with antibody labeling

Despite extensive research on MG in social insects (for a review, see Fahrbach and Van Nest 2016), relatively little is known about the PN-KC connectivity so far. MB KCs in honeybees can be subdivided into two classes based on their morphology: class I KCs show a very dense arborization pattern and exhibit thin, spine-like dendritic specializations, while class II KCs form large claw-like endings potentially providing multiple contacts to a single PN bouton (Mobbs 1982; Strausfeld 2002; for reviews see: Fahrbach 2006; Groh and Rössler 2020). The majority of KCs in the honeybee MB are class I KCs, which restrict their dendritic arborizations in the MB calyx to either the olfactory lip, the visual collar, or the bi-modal basal ring (Strausfeld 2002). Class II KCs, on the other hand, may extend their dendrites across all three calyx subcompartments and might thus potentially receive multimodal input. The PN-KC connectivity and structural details of dendritic specializations are not classified in detail (discussed in Groh and Rössler 2020). In *D. melanogaster*, the MB consists only of clawed KCs and detailed knowledge about their structure and connectivity was provided by using genetic tools (e.g., Leiss et al. 2009; Baltruschat et al. 2021). In the honeybee and most other insects, these tools are not available. Therefore, neuronal tracings using dye injection are a widely used tool for single cell and mass labeling. However, at the level of individual dendritic spines or fine branches, restrictions are posed by the diffraction limit of conventional confocal laser scanning microscopy. Combining ExM with neuronal tracings and antibody labeling provides a method that allows new insights into the pre- and postsynaptic architecture of MG.

Post-labeling of expanded specimen with fluorescently labeled streptavidin achieved a good overall staining quality of tracings revealing fine details of arborizations. Olfactory PN boutons appeared in very different outer shapes and sizes, which coincides with previous findings in the MB lip based on serial-section EM (Groh et al. 2012). Whether these differences in structure cause functional differences needs to be tested by neurophysiological methods. A very interesting observation is the heterogenous distribution of vesicle clusters at different PN boutons. While some boutons exhibited a very high density of synapsin-IR clusters in their center and at their outer membrane (Fig. 8c), in other boutons, synapsin clusters were only sparsely distributed or not existent at all (Fig. 8b). This correlates with differences in cytoplasmic electron densities within PN boutons observed with serial-section EM, which likely represents differences in the abundance of small clear-core vesicles (Groh et al. 2012). Following ideas of Groh et al. (2012) and an early EM study in ants by Steiger (1967), we hypothesize that differences in the number of synaptic vesicles in PN boutons might reflect different functional states of PNs. For instance, it seems likely that boutons with a low number of synaptic vesicles are either immature or in a quiescent state (“sleeping synapses”) until activation through learning processes or changes in sensory environment. In fact, Butcher et al. (2012) showed that dark boutons in MB calyces of *Drosophila* had a higher density of ribbon synapses, which are presumed to represent readily activated synapses enabling fast transmission. Silencing of PNs, on the other hand, leads to an increase of BRP puncta per PN bouton, and thus an increase in AZs, in *Drosophila* (Kremer et al. 2010). The functional implications of variances in synaptic vesicle density are therefore not fully understood. By combining neuronal tracing of PNs with synapsin immunostaining and subsequent ExM, the increased resolution allows for identification and quantification of vesicle clusters at individual PN boutons. This provides a new avenue to assess synaptic plasticity in response to variable external and internal stimuli.

In another approach, we successfully retrogradely traced a population of class I KCs back from their terminal projections in the vertical lobe into the basal ring region. The classification to this group was determined via the position of dye injection (Fig. 8d, inset I) and the position of stained KC somata (Fig. 8d, inset II). In combination with the presynaptic markers synapsin and BRP, we were able to visualize the pre- and postsynaptic architecture of MG in the MB calyces at high resolution. The amount of detail even allows for identification of putative synaptic contacts, which has never been provided for honeybees at the light microscopic level before (see inset in Fig. 8f for a corresponding electron microscopic image). In contrast to our expectation, the dendritic specializations of the labeled class I KCs did not appear as thin, distinct spines protruding from the dendrites as shown in previous Golgi preparations (Strausfeld 2002), but were rather entangled in a dense dendritic web. As multiple cells were stained, it is not possible to break down the structure and number of contacts to an individual KC. Nevertheless, this method has a high potential for future analyses of MG, for example, labeling of small KC numbers or even individual KCs using electroporation, iontophoretic, or pressure injections. Furthermore, PN and KC tracings can be combined with anti-synapsin and -BRP labeling to analyze multiple parameters as a proxy for plastic changes at these synaptic complexes in different KC classes.

Altogether, we conclude that (pro-)ExM presents a valuable and highly promising technique that can readily be used in insects in combination with immunolabeling and neuronal tracing techniques. With an increasing number of antibodies becoming available in the honeybee, the use of ExM opens an important avenue for high-resolution analyses of neuronal and synaptic structures at subcellular levels related to neuronal plasticity (Hurd et al. 2021).

## Data Availability

All relevant data are included in the manuscript. Raw data can be made available by the author upon reasonable request.

## References

[CR1] Adler J, Parmryd I (2010). Quantifying colocalization by correlation: the Pearson correlation coefficient is superior to the Mander’s overlap coefficient. Cytom Part A.

[CR2] Asano SM, Gao R, Wassie AT et al (2018) Expansion Microscopy: protocols for imaging proteins and RNA in cells and tissues. Curr Protoc Cell Biol 80:e56. 10.1002/cpcb.56 10.1002/cpcb.56 PMC6158110 30070431

[CR3] Baltruschat L, Prisco L, Ranft P et al (2021) Circuit reorganization in the *Drosophila* mushroom body calyx accompanies memory consolidation. Cell Rep 34:108871. 10.1016/j.celrep.2021.108871 10.1016/j.celrep.2021.108871 PMC8515896 33730583

[CR4] Blenau W, Schmidt M, Faensen D, Schürmann FW (1999). Neurons with dopamine-like immunoreactivity target mushroom body Kenyon cell somata in the brain of some hymenopteran insects. Int J Insect Morphol Embryol.

[CR5] Butcher NJ, Friedrich AB, Lu Z (2012). Different classes of input and output neurons reveal new features in microglomeruli of the adult *Drosophila* mushroom body calyx. J Comp Neurol.

[CR6] Cabirol A, Brooks R, Groh C (2017). Experience during early adulthood shapes the learning capacities and the number of synaptic boutons in the mushroom bodies of honey bees (*Apis mellifera*). Learn Mem.

[CR7] Cesca F, Baldelli P, Valtorta F, Benfenati F (2010). The synapsins: Key actors of synapse function and plasticity. Prog Neurobiol.

[CR8] Chang J-B, Chen F, Yoon Y-G (2017). Iterative expansion microscopy. Nat Methods.

[CR9] Chen F, Tillberg PW, Boyden ES (2015). Expansion microscopy. Science.

[CR10] Chen F, Wassie AT, Cote AJ (2016). Nanoscale imaging of RNA with expansion microscopy. Nat Methods.

[CR11] Durst C, Eichmüller S, Menzel R (1994). Development and experience lead to increased volume of subcompartments of the honeybee mushroom body. Behav Neural Biol.

[CR12] Fahrbach SE (2006). Structure of the mushroom bodies of the insect brain. Annu Rev Entomol.

[CR13] Fahrbach SE, Van Nest BN (2016). Synapsin-based approaches to brain plasticity in adult social insects. Curr Opin Insect Sci.

[CR14] Fahrbach SE, Moore D, Capaldi EA (1998). Experience-expectant plasticity in the mushroom bodies of the honeybee. Learn Mem.

[CR15] Falibene A, Roces F, Rössler W (2015). Long-term avoidance memory formation is associated with a transient increase in mushroom body synaptic complexes in leaf-cutting ants. Front Behav Neurosci.

[CR16] Forte N, Binda F, Contestabile A (2020). Synapsin I Synchronizes GABA Release in Distinct Interneuron Subpopulations. Cereb Cortex.

[CR17] Fouquet W, Owald D, Wichmann C (2009). Maturation of active zone assembly by *Drosophila* Bruchpilot. J Cell Biol.

[CR18] Frambach I, Rössler W, Winkler M, Schürmann FW (2004). F-actin at identified synapses in the mushroom body neuropil of the insect brain. J Comp Neurol.

[CR19] Gambarotto D, Hamel V, Guichard P (2021). Ultrastructure expansion microscopy (U-ExM). Methods Cell Biol.

[CR20] Ganeshina O, Menzel R (2001). GABA-immunoreactive neurons in the mushroom bodies of the honeybee: an electron microscopic study. J Comp Neurol.

[CR21] Gao R, Asano SM, Boyden ES (2017). Q&A: Expansion microscopy. BMC Biol.

[CR22] Gao M, Maraspini R, Beutel O (2018). Expansion stimulated emission depletion microscopy (ExSTED). ACS Nano.

[CR23] Gehring KB, Heufelder K, Depner H et al (2017) Age-associated increase of the active zone protein Bruchpilot within the honeybee mushroom body. PLoS One 12:e0175894. 10.1371/journal.pone.0175894 10.1371/journal.pone.0175894 PMC5402947 28437454

[CR24] Giurfa M (2007). Behavioral and neural analysis of associative learning in the honeybee: a taste from the magic well. J Comp Physiol A.

[CR25] Giurfa M (2013). Cognition with few neurons: higher-order learning in insects. Trends Neurosci.

[CR26] Götz R, Panzer S, Trinks N (2020). Expansion microscopy for cell biology analysis in fungi. Front Microbiol.

[CR27] Greengard P, Valtorta F, Czernik AJ, Benfenati F (1993). Synaptic vesicle phosphoproteins and regulation of synaptic function. Science.

[CR28] Groh C, Rössler W (2020). Analysis of synaptic microcircuits in the mushroom bodies of the honeybee. Insects.

[CR29] Groh C, Ahrens D, Rössler W (2006). Environment- and age-dependent plasticity of synaptic complexes in the mushroom bodies of honeybee queens. Brain Behav Evol.

[CR30] Groh C, Lu Z, Meinertzhagen IA, Rössler W (2012). Age-related plasticity in the synaptic ultrastructure of neurons in the mushroom body calyx of the adult honeybee *Apis mellifera*. J Comp Neurol.

[CR31] Gronenberg W (2001). Subdivisions of hymenopteran mushroom body calyces by their afferent supply. J Comp Neurol.

[CR32] Grünewald B (1999). Morphology of feedback neurons in the mushroom body of the honeybee, *Apis mellifera*. J Comp Neurol.

[CR33] Grünewald B (1999). Physiological properties and response modulations of mushroom body feedback neurons during olfactory learning in the honeybee, *Apis mellifera*. J Comp Physiol - A Sensory, Neural, Behav Physiol.

[CR34] Haehnel M, Menzel R (2010). Sensory representation and learning-related plasticity in mushroom body extrinsic feedback neurons of the protocerebral tract. Front Syst Neurosci.

[CR35] Haehnel M, Menzel R (2012). Long-term memory and response generalization in mushroom body extrinsic neurons in the honeybee *Apis mellifera*. J Exp Biol.

[CR36] Hammer M (1993). An identified neuron mediates the unconditioned stimulus in associative olfactory learning in honeybees. Nature.

[CR37] Heisenberg M (1998). What do the mushroom bodies do for the insect brain? An introduction. Learn Mem.

[CR38] Hourcade B, Muenz TS, Sandoz JC (2010). Long-term memory leads to synaptic reorganization in the mushroom bodies: A memory trace in the insect brain?. J Neurosci.

[CR39] Hurd PJ, Grübel K, Wojciechowski M (2021). Novel structure in the nuclei of honey bee brain neurons revealed by immunostaining. Sci Rep.

[CR40] Jiang N, Kim H-J, Chozinski TJ (2018). Superresolution imaging of *Drosophila* tissues using expansion microscopy. Mol Biol Cell.

[CR41] Kassambara A (2020) ggpubr: “ggplot2” Based Publication Ready Plots. R package version 0.4.0. https://CRAN.R-project.org/package=ggpubr

[CR42] Kittel RJ, Heckmann M (2016). Synaptic vesicle proteins and active zone plasticity. Front Synaptic Neurosci.

[CR43] Kittel RJ, Wichmann C, Rasse TM (2006). Bruchpilot promotes active zone assembly, Ca^2+^ channel clustering, and vesicle release. Science.

[CR44] Klagges BRE, Heimbeck G, Godenschwege TA (1996). Invertebrate synapsins: A single gene codes for several isoforms in *Drosophila*. J Neurosci.

[CR45] Kremer MC, Christiansen F, Leiss F (2010). Structural Long-Term Changes at Mushroom Body Input Synapses. Curr Biol.

[CR46] Kunz TC, Götz R, Gao S (2020). Using Expansion Microscopy to Visualize and Characterize the Morphology of Mitochondrial Cristae. Front Cell Dev Biol.

[CR47] Leiss F, Groh C, Butcher NJ (2009). Synaptic organization in the adult *Drosophila* mushroom body calyx. J Comp Neurol.

[CR48] Markert SM, Bauer V, Muenz TS (2017). 3D subcellular localization with superresolution array tomography on ultrathin sections of various species. Methods Cell Biol.

[CR49] Marstal K, Berendsen F, Staring M, Klein S (2016) SimpleElastix: a user-friendly, multi-lingual library for medical image registration. In: 2016 IEEE Conference on Computer Vision and Pattern Recognition Workshops (CVPRW). pp 574–582

[CR50] Menzel R (1999). Memory dynamics in the honeybee. J Comp Physiol - A Sensory, Neural, Behav Physiol.

[CR51] Mobbs PG (1982). The brain of the honeybee *Apis mellifera*. I. The connections and spatial organization of the mushroom bodies. Philos Trans R Soc London B, Biol Sci.

[CR52] Muenz TS, Groh C, Maisonnasse A (2015). Neuronal plasticity in the mushroom body calyx during adult maturation in the honeybee and possible pheromonal influences. Dev Neurobiol.

[CR53] Okada R, Rybak J, Manz G, Menzel R (2007) Learning-Related Plasticity in PE1 and Other Mushroom Body-Extrinsic Neurons in the Honeybee Brain. J Neurosci 27:11736 LP – 11747. 10.1523/JNEUROSCI.2216-07.2007 10.1523/JNEUROSCI.2216-07.2007 PMC6673233 17959815

[CR54] Prisco L, Deimel SH, Yeliseyeva H et al (2021) The anterior paired lateral neuron normalizes odour-evoked activity in the *Drosophila* mushroom body calyx. Elife 10:e74172. 10.7554/eLife.74172 10.7554/eLife.74172 PMC8741211 34964714

[CR55] Raccuglia D, Mueller U (2013). Focal uncaging of GABA reveals a temporally defined role for GABAergic inhibition during appetitive associative olfactory conditioning in honeybees. Learn Mem.

[CR56] Rybak J, Menzel R (1993). Anatomy of the mushroom bodies in the honey bee brain: the neuronal connections of the alpha-lobe. J Comp Neurol.

[CR57] Sadanandappa MK, Redondo BB, Michels B et al (2013) Synapsin Function in GABA-ergic Interneurons Is Required for Short-Term Olfactory Habituation. J Neurosci 33:16576 – 16585. 10.1523/JNEUROSCI.3142-13.2013 10.1523/JNEUROSCI.3142-13.2013 PMC6618520 24133261

[CR58] Schäfer S, Bicker G (1986). Distribution of GABA-like immunoreactivity in the brain of the honeybee. J Comp Neurol.

[CR59] Schindelin J, Arganda-Carreras I, Frise E (2012). Fiji: An open-source platform for biological-image analysis. Nat Methods.

[CR60] Scholl C, Wang Y, Krischke M (2014). Light exposure leads to reorganization of microglomeruli in the mushroom bodies and influences juvenile hormone levels in the honeybee. Dev Neurobiol.

[CR61] Schürmann FW (2016). Fine structure of synaptic sites and circuits in mushroom bodies of insect brains. Arthropod Struct Dev.

[CR62] Song S-H, Augustine GJ (2016) Synapsin Isoforms Regulating GABA Release from Hippocampal Interneurons. J Neurosci 36:6742 LP – 6757. 10.1523/JNEUROSCI.0011-16.2016 10.1523/JNEUROSCI.0011-16.2016 PMC6601744 27335405

[CR63] Steiger U (1967). Über den Feinbau des Neuropils im Corpus pedunculatum der Waldameise - Elektronenoptische Untersuchungen. Zeitschrift Für Zellforsch Und Mikroskopische Anat.

[CR64] Strausfeld NJ (2002). Organization of the honey bee mushroom body: Representation of the calyx within the vertical and gamma lobes. J Comp Neurol.

[CR65] Sun De-en, Fan X, Shi Y (2021). Click-ExM enables expansion microscopy for all biomolecules. Nat Methods.

[CR66] Tillberg PW, Chen F, Piatkevich KD (2016). Protein-retention expansion microscopy of cells and tissues labeled using standard fluorescent proteins and antibodies. Nat Biotechnol.

[CR67] Trinks N, Reinhard S, Drobny M et al (2021) Subdiffraction-resolution fluorescence imaging of immunological synapse formation between NK cells and *A. fumigatus* by expansion microscopy. Commun Biol 4:1151. 10.1038/s42003-021-02669-y 10.1038/s42003-021-02669-y PMC8490467 34608260

[CR68] Ullrich A, Böhme MA, Schöneberg J et al (2015) Dynamical Organization of Syntaxin-1A at the Presynaptic Active Zone. PLoS Comput Biol 11:e1004407. 10.1371/journal.pcbi.1004407 10.1371/journal.pcbi.1004407 PMC4569342 26367029

[CR69] Wagh DA, Rasse TM, Asan E (2006). Bruchpilot, a protein with homology to ELKS/CAST, is required for structural integrity and function of synaptic active zones in *Drosophila*. Neuron.

[CR70] Wang Y, Yu Z, Cahoon CK (2018). Combined expansion microscopy with structured illumination microscopy for analyzing protein complexes. Nat Protoc.

[CR71] Wassie AT, Zhao Y, Boyden ES (2019). Expansion microscopy: principles and uses in biological research. Nat Methods.

[CR72] Wichmann C, Sigrist SJ (2010). The active zone T-bar - A plasticity module?. J Neurogenet.

[CR73] Withers GS, Fahrbach SE, Robinson GE (1993). Selective neuroanatomical plasticity and division of labour in the honeybee. Nature.

[CR74] Yu CC, Barry NC, Wassie AT et al (2020) Expansion Microscopy of c Elegans Elife 9:1–78. 10.7554/ELIFE.46249 10.7554/eLife.46249 PMC7195193 32356725

[CR75] Zhao Y, Bucur O, Irshad H (2017). Nanoscale imaging of clinical specimens using pathology-optimized expansion microscopy. Nat Biotechnol.

[CR76] Zwaka H, Bartels R, Grünewald B, Menzel R (2018). Neural Organization of A3 Mushroom Body Extrinsic Neurons in the Honeybee Brain. Front Neuroanat.

[CR77] Zwettler FU, Reinhard S, Gambarotto D (2020). Molecular resolution imaging by post-labeling expansion single-molecule localization microscopy (Ex-SMLM). Nat Commun.

